# Instrumentation Digital Twins in PET and SPECT Imaging: Current Status, Challenges, and Future Directions

**DOI:** 10.34133/csbj.0046

**Published:** 2026-04-09

**Authors:** Mohammad Saber Azimi, Nicolas A. Karakatsanis, Hamid Sabet, Magdalena Rafecas, Sadek Nehmeh, Abass Alavi, Michael King, Habib Zaidi

**Affiliations:** ^1^ Doctoral School of Applied Informatics and Applied Mathematics, Óbuda University, Budapest, Hungary.; ^2^ University Research and Innovation Center, Óbuda University, Budapest, Hungary.; ^3^ Department of Radiology, Weill Cornell Medicine, New York, NY, USA.; ^4^ Department of Radiology, Massachusetts General Hospital, Harvard Medical School, Boston, MA, USA.; ^5^ Institute of Medical Engineering, University of Lübeck, Lübeck, Germany.; ^6^ Department of Radiology, Perelman School of Medicine, University of Pennsylvania, Philadelphia, PA, USA.; ^7^ Department of Radiology, University of Massachusetts Chan Medical School, Worcester, MA, USA.; ^8^ Division of Nuclear Medicine & Molecular Imaging, Geneva University Hospital, CH-1211 Geneva, Switzerland.; ^9^ Department of Nuclear Medicine and Molecular Imaging, University of Groningen, University Medical Center Groningen, Groningen, Netherlands.; ^10^ Department of Nuclear Medicine, University of Southern Denmark, Odense, Denmark.

## Abstract

**Introduction:** Digital twins (DTs) in nuclear medicine are data-synchronized, physics-based models that replicate positron emission tomography (PET) and single-photon emission computed tomography (SPECT) systems by linking validated simulations with scanner configuration and calibration inputs. Instrumentation DTs (iDTs) extend system modeling by enabling feedback-driven assessment of detector performance, collimation, electronics behavior, and acquisition conditions. This review summarizes recent developments in PET and SPECT iDTs and evaluates their roles in system optimization. **Materials and Methods:** A structured literature review was conducted, and applications were grouped into 6 domains: (a) calibration and performance optimization, (b) predictive maintenance and fault detection, (c) quantitative correction and protocol optimization, (d) dosimetry and reconstruction benchmarking, (e) synthetic data generation and artificial intelligence (AI) training, and (f) scanner design and geometry evaluation. Attention was given to implementations integrating established detector and system models into unified, data-linked workflows. **Results:** Evidence shows that iDTs support virtual calibration, protocol assessment, and algorithm benchmarking. They can simulate detector misalignment and probabilistic hardware failures, factors relevant for high-resolution and small-animal imaging. iDTs also enable exploration of geometry changes, dose-sensitivity trade-offs, and long axial field-of-view configurations. However, current approaches remain fragmented, depend on vendor-specific components, and provide limited telemetry integration. Validation still relies mainly on standard National Electrical Manufacturers Association and quality control procedures. **Conclusion:** Future progress will benefit from modular and standardized iDT frameworks incorporating hardware realism, reliability modeling, and hybrid AI–physics strategies. Fully developed iDTs can improve calibration stability, support adaptive protocol design, enable early detection of component degradation, guide dose optimization, and accelerate virtual prototyping of next-generation PET and SPECT systems.

## Graphical Abstract

**Figure:**
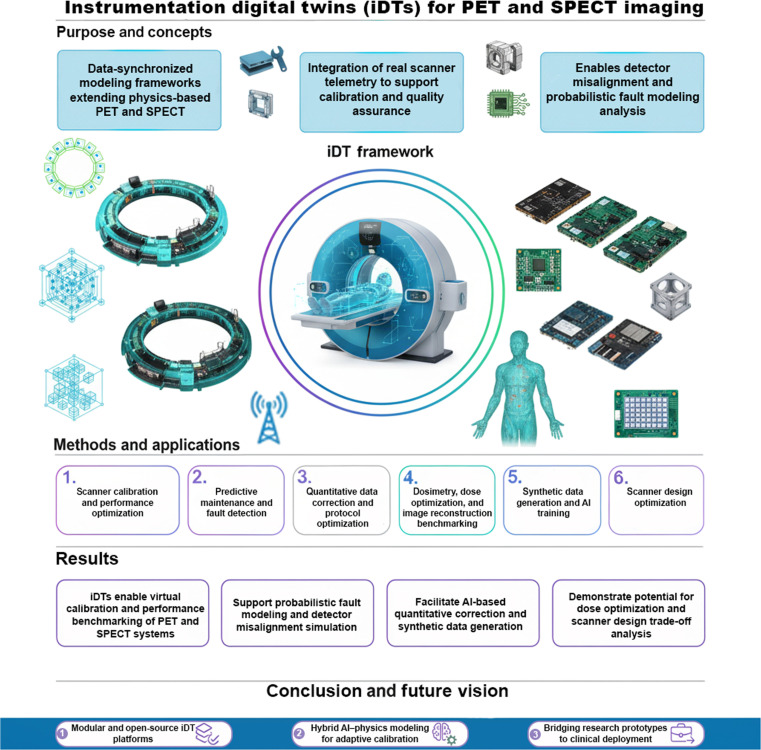

## Introduction

The concept of the digital twin (DT), a dynamic digital replica of a physical system, has transformed multiple industries by enabling real-time simulation, monitoring, and optimization of complex processes [1,2]. In healthcare, DT technology is increasingly applied to patient-specific modeling, treatment planning, and personalized medicine [3,4]. While the majority of DT research in medicine has been focused on patient-specific modeling and therapy planning [5–7], a rapidly growing subfield explores the use of DTs to model and simulate the behavior of medical imaging instruments themselves [8–10]. In this review, we use the term instrumentation digital twin (iDT) to refer to a data-synchronized, simulation-based digital replica of a positron emission tomography (PET) or single-photon emission computed tomography (SPECT) scanner that captures hardware-level behavior, including collimators, detectors, electronics chains, and calibration characteristics, and that can update its parameters using real or simulated acquisition data. Within the broader DT taxonomy, iDTs represent the scanner-centric subclass of DTs. Unlike static or stand-alone simulations, iDTs integrate telemetry and model hardware performance under realistic operating conditions, and support iterative or bidirectional updates between the physical system and its digital counterpart. Accordingly, studies describing advanced detector technologies, Monte Carlo (MC) simulation frameworks, or system modeling approaches are discussed in this review as enabling components that contribute to the development of iDT frameworks rather than as stand-alone DTs. Throughout this manuscript, all references to DTs specifically denote iDTs unless otherwise noted. Current implementations in nuclear imaging span multiple stages of DT maturity along a developmental continuum, ranging from physics-based simulation environments and validated digital replicas to emerging adaptive DT frameworks capable of integrating system telemetry.

In nuclear medicine, where quantitative accuracy and image quality depend heavily on detector physics, system calibration, and patient motion, iDTs offer unique opportunities. By integrating physics-based modeling (e.g., MC simulations), system-specific parameters, and reconstruction pipelines, digital replicas—or DTs—of PET and SPECT scanners can be developed and used for in silico experimentation, scan protocol optimization, predictive maintenance, validation of image generation and analysis methods, and improved training and validation of artificial intelligence (AI)-powered algorithms [3,11–18]. These digital instrumentation replicas integrate physics-based modeling (e.g., MC simulations), system-specific configurations, and hardware and software environments to emulate the scanner's real-world behavior under diverse operational conditions [19,20]. Tools, such as Geant4 Application for Tomographic Emission (GATE), have become central to iDT modeling, enabling realistic simulation of PET and SPECT systems with high detector- and geometry-specific fidelity [21,22]. Moreover, when integrated with digital anthropomorphic patient models, such as the XCAT phantom [23,24], iDT frameworks enable clinically realistic investigations of organ motion, tracer kinetics, and scanner–patient interactions under controlled and reproducible conditions.

Recent advances in both PET and SPECT technologies, such as long axial field-of-view (LAFOV) PET/computed tomography (CT), solid-stage (CZT)-based SPECT detectors, and resolution-recovery reconstruction algorithms, provide a strong foundation for developing scanner-centric DT frameworks [25,26]. Several studies have already reported on DTs of commercial PET scanners, for example, the Siemens Biograph Vision Quadra, validated against experimental measurements and applied to motion correction, dosimetry, and algorithm benchmarking [8,27–29]. These iDT frameworks enable researchers and clinicians to study scanner behavior under various controlled clinical and experimental conditions without relying solely on physical systems [30]. They also facilitate validation of quantitative data correction frameworks, such as motion correction techniques [31], determination of optimal administered activity [32,33], estimation of absorbed dose distributions [34], benchmarking of image reconstruction algorithms [35], and generation of synthetic datasets for training deep learning models [36].

Despite these advances, there is no unified framework that consolidates iDT applications in PET and SPECT into a structured roadmap. Most prior research has focused on isolated aspects (e.g., motion correction, dose estimation, or AI training), leaving an unmet need for comprehensive analysis. This review addresses that gap by:

1. Summarizing the current status of iDTs in nuclear medicine imaging;
2. Identifying key challenges in data integration, modeling, and clinical translation; and
3. Outlining a roadmap for future directions, including opportunities for AI integration, real-time system monitoring, and clinical adoption.

Through this lens, the paper asks a central question: Can iDTs evolve from in silico research prototypes into a foundational infrastructure for smarter, more adaptive PET and SPECT systems?

The literature included in this review was identified through searches of major scientific databases including PubMed, Scopus, and Web of Science. The search strategy used combinations of keywords including “digital twin”, “medical imaging”, “nuclear medicine”, “PET”, “SPECT”, “instrumentation”, “Monte Carlo simulation”, “detector modeling”, “image reconstruction”, “dosimetry”, “artificial intelligence”, “predictive maintenance”, and “federated learning”. Studies were selected based on their relevance to the development, implementation, or application of DT concepts in medical imaging instrumentation, imaging workflows, or patient-specific modeling. Both methodological developments and application-oriented studies were considered. After screening titles, abstracts, and full texts, a total of 236 relevant publications were included in this review.

Following this introduction, the “Background and Concepts” section introduces the background and key concepts, including the definition of DTs in healthcare and the fundamental principles of PET and SPECT imaging. The “State of the Art in iDT for Medical Imaging” section then presents a structured review of iDT applications across 6 domains: scanner calibration and performance optimization, predictive maintenance and fault detection, quantitative data correction and protocol optimization, dosimetry and reconstruction benchmarking, synthetic data generation and AI training, and scanner design optimization. The “Challenges in iDTs for PET and SPECT Imaging” section discusses the major challenges and limitations of current iDT implementations, while the “Roadmap and Future Directions” section outlines a roadmap for future development, emphasizing hybrid AI–physics models, real-time feedback, and clinical integration. Comparative tables in these sections provide concise summaries of representative studies, highlighting both achievements and remaining gaps. Finally, the “Conclusion” section reflects on the overall role of iDTs in advancing PET and SPECT instrumentation and their prospects for clinical adoption.

## Background and Concepts

### DTs in healthcare

A DT in healthcare is commonly defined as a virtual counterpart of a biological system or medical device that can be continuously updated with real-world data [37]. The following are 3 defining features:

1. Real-time synchronization between the physical and digital entities [38];
2. Bidirectional data flow, allowing insights from the DT to inform the physical system [39]; and
3. Predictive capability, where simulations anticipate system performance and patient outcomes [40].

Beyond nuclear medicine, DTs have been increasingly applied in radiology. A recent systematic review by Faiella et al. [4] identified applications across abdominal, musculoskeletal, interventional, dental, cardiothoracic, and neuroimaging domains. Recent studies have also explored immersive virtual-reality environments as patient-specific DTs for surgical planning and anatomical assessment, demonstrating improved spatial understanding and diagnostic accuracy in complex thoracic malformations [41]. While patient-specific DTs have shown benefits in disease modeling and therapy optimization, iDTs offer complementary advantages, such as improved system calibration, quantitative accuracy, and image quality enhancement, highlighting iDTs as an emerging framework that integrates computational modeling, multimodal imaging, and AI for more reliable nuclear medicine instrumentation.

### PET and SPECT imaging

PET and SPECT are key nuclear medicine imaging modalities that visualize molecular processes in vivo through the biodistribution of radiotracers. In PET, positron-emitting radiopharmaceuticals undergo β^+^ decay followed by positron–electron annihilation, producing pairs of 511-keV photons detected in coincidence, enabling high sensitivity and quantitative imaging [42,43]. SPECT acquires single gamma photons using collimators and gamma cameras and more recently solid-state (CZT)-based devices, and benefits from the widespread availability and lower production cost of radiotracers, such as those labeled with ^99m^Tc [25,26,44].

Despite their clinical importance, both modalities face persistent limitations:

• Detector sensitivity and spatial plus energy resolution constrain image quality;
• Physical effects, such as scatter and attenuation, degrade quantification and can cause artifacts in the images;
• Patient motion introduces additional degradation of spatial resolution plus artifacts; and
• Calibration and reconstruction protocols vary between systems [45,46].
• In PET, certain emerging radiotracers emit prompt or cascade gamma rays, increasing randoms and scatter and complicating quantitative accuracy.
• In SPECT, radiotracers with multienergy photon emissions challenge system performance, since collimators are optimized for a specific energy range.

**Fig. 1.**
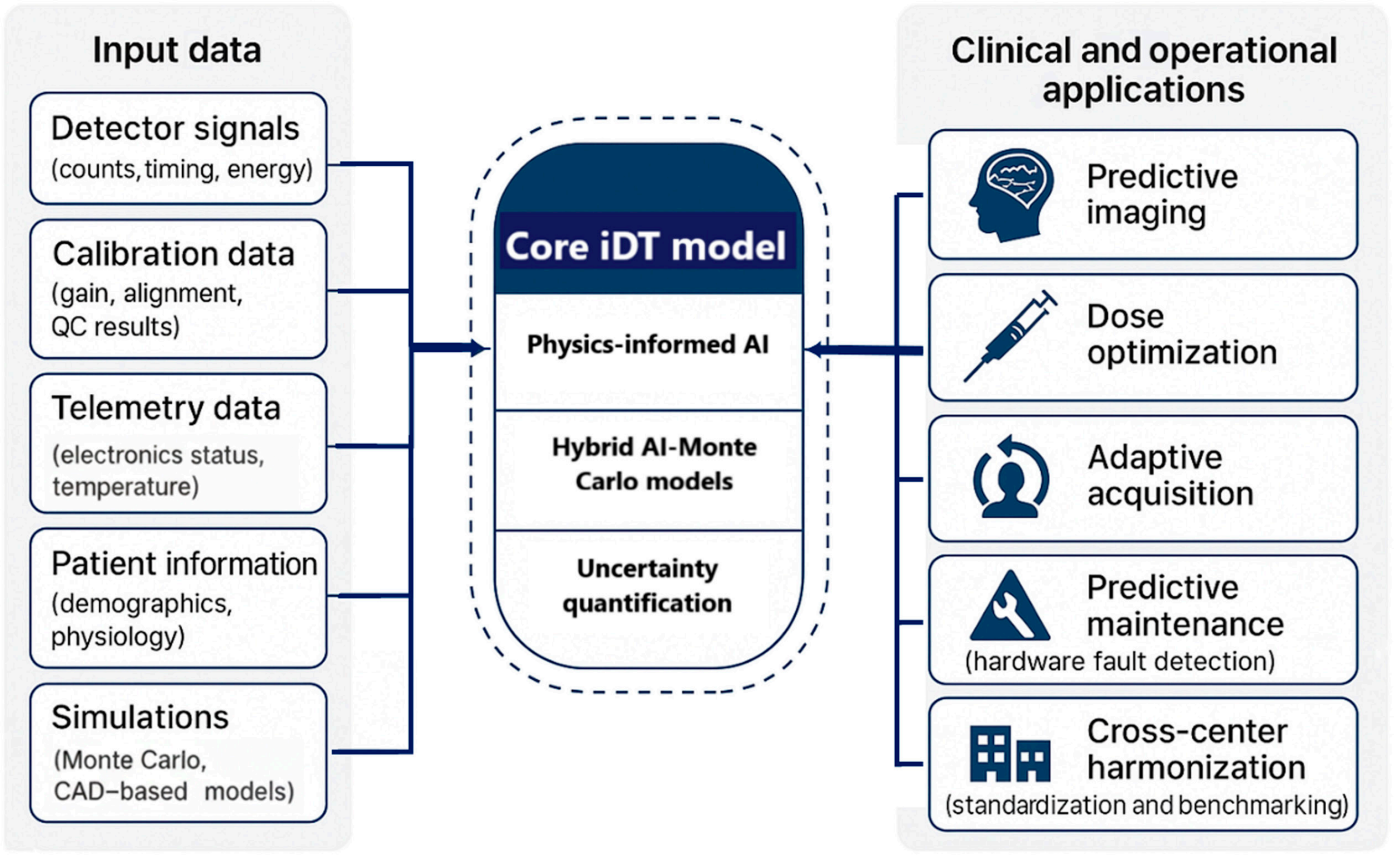
Conceptual architecture of instrumentation digital twins (iDTs) for positron emission tomography (PET) and single-photon emission computed tomography (SPECT) imaging. Input data streams, including detector signals, calibration and telemetry data, patient information, and digital phantoms, are integrated into a hybrid digital twin (DT) model combining physics-based simulations and artificial intelligence (AI) surrogates. The resulting iDT enables a wide spectrum of clinical and operational applications, ranging from predictive imaging and adaptive acquisition to dose optimization, predictive maintenance, and cross-center harmonization.

These challenges create a clear rationale for digital replicas of scanners that can simulate detector physics, acquisition protocols, and reconstruction pipelines. iDTs offer a structured way to investigate and potentially reduce the intrinsic limitations of PET and SPECT systems. As illustrated in Fig. 1, DTs bridge the physical scanner and the clinical workflow through physics-informed simulations and AI models.

## State of the Art in iDT for Medical Imaging

### Lessons from DT applications in other imaging modalities

While this review primarily focuses on PET and SPECT systems, insights from other imaging modalities can help illustrate how iDT frameworks have been utilized to improve system understanding and workflow optimization [47]. In CT and MRI, iDTs have been applied for acquisition protocol modeling, virtual hardware optimization, quantitative data correction, and reconstruction algorithm development plus validation [48]. For instance, in MRI, Wang et al. [49] demonstrated a multimodal iDT framework enhanced by deep transfer learning, enabling super-resolution image reconstruction and adaptive fusion of MRI with PET or SPECT data, thereby improving diagnostic fidelity and artifact suppression. In CT, Dahal et al. [50] introduced XCAT 3.0, a comprehensive library of over 2,500 personalized anthropomorphic DTs derived from full-body CT scans, which supports virtual imaging trials and protocol harmonization by providing anatomically detailed computational phantoms across diverse patient populations. Similar initiatives in ultrasound imaging have applied iDT frameworks to improve probe calibration, compensate for motion artifacts, and enhance operator training, as demonstrated by Dang et al. [51], who developed a DT-based interactive device that enables efficient and cost-effective simulation of scanning tasks. Collectively, these examples highlight how DTs extend beyond patient-level modeling toward system-level applications in diverse imaging modalities.

### Nuclear medicine: PET and SPECT imaging

**Fig. 2.**
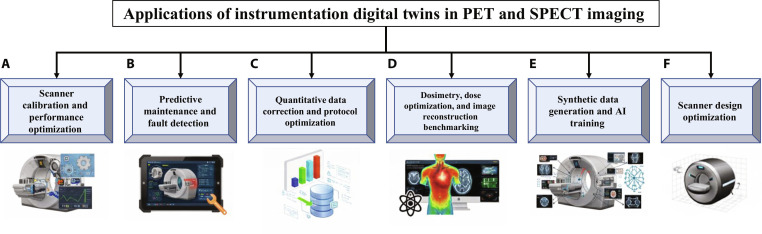
Classification of major application domains of iDTs in PET and SPECT imaging systems. The framework categorizes DT use cases into 6 major domains: (A) scanner calibration and performance optimization; (B) predictive maintenance and fault detection; (C) quantitative data correction and protocol optimization; (D) dosimetry, dose optimization, and image reconstruction benchmarking; (E) synthetic data generation and AI training; and (F) scanner design optimization. These categories reflect how DTs are being leveraged to address scanner-level challenges, from hardware calibration to data-driven model development, highlighting their potential to enhance reliability, reproducibility, and clinical translation in nuclear imaging.

Compared with CT or MRI, iDT frameworks in nuclear medicine have been predominantly oriented toward the hardware level, where scanner performance depends critically on detector physics, electronics, and system geometry. Most existing works explored isolated applications, such as calibration, failure detection, or quantitative reconstruction benchmarking, rather than unified iDT frameworks including all components of an imaging experiment from instrumentation to imaging pipelines and clinical applications. Conceptually, iDTs extend the well-established MC simulation paradigm by integrating detailed hardware models with real or synthetic operational data. MC simulations remain the core engine behind most iDT implementations, with widely used software platforms, such as GATE, SIMIND, MCNP, Geant4, and other packages, providing physics-accurate modeling of photon transport and detector interactions; recent graphics processing unit (GPU)-accelerated MC frameworks further enable substantial speed-ups that make near-real-time or large-scale iDT applications increasingly feasible [52–56]. Unlike conventional simulations that represent static systems, iDTs are dynamic frameworks capable of continuous calibration, adaptive parameter tuning, and predictive analysis across scanner components. Below, we outline the principal hardware-oriented application domains, highlighting representative advances and persisting challenges. These iDT applications can be broadly classified into 6 domains (Fig. 2), which are discussed in the following subsections.

#### Scanner calibration and performance optimization

Hardware-level iDTs of PET and SPECT scanners support calibration workflows by simulating detector gain, timing alignment, and spatial positioning under controlled conditions, thereby complementing and informing the calibration of physical systems. For LAFOV PET systems, such as the PennPET Explorer and uEXPLORER, iDT-based simulations have been used to study effects of detector gaps, normalization accuracy, and dead-time correction under extended geometries [57–60]. In small-animal PET, DTs incorporating monolithic scintillators or depth-of-interaction (DOI) detectors allow designs with submillimeter resolution considering parallax error [61–65]. Modeling such systems often requires accurate representation of scintillation light transport, particularly for long crystals and shared-light readout geometries. Conventional MC photon-tracking can become prohibitively time consuming for full acquisitions, and hybrid approaches have been proposed to address this limitation. Solevi et al. developed a computational model of optical-photon propagation for the AX-PET demonstrator, demonstrating how such hybrid optical modeling can complement GATE simulations and improve the fidelity of DTs for monolithic or light-sharing PET designs [66]. Such simulations provide insight into calibration strategies that are challenging to validate experimentally, ensuring stable performance across different hardware configurations [67].

Parallel advances in SPECT calibration have focused on collimator optimization, detector uniformity, and resolution-recovery reconstruction methods [68–74]. Here, iDT frameworks provide a virtual environment for testing collimator designs, modeling system sensitivity trade-offs, and harmonizing calibration across centers. These PET and SPECT examples collectively highlight how iDT-based calibration and performance optimization can address both technical and clinical workflow challenges.

#### Predictive maintenance and fault detection

Borrowing from industrial reliability engineering, iDTs can be used to model failure probabilities of hardware components such as photomultiplier tubes (PMTs), silicon photomultipliers (SiPMs), scintillators, and movable (i.e. translating or rotating) gantry elements [75]. Approaches based on probabilistic safety assessment (PSA), including event-tree and fault-tree analysis, have already been explored in movable machinery and industrial Internet of Things (IoT) environments, showing potential for fault localization and decentralized monitoring [76,77]. Extending these concepts to nuclear imaging, iDT frameworks could simulate gain drift in scintillators or PMTs, thermal imbalance in electronics, and motor wear in rotating SPECT gantries, allowing early prediction of degradation trajectories.

Emerging AI-enhanced iDT approaches in nuclear imaging also draw inspiration from predictive-maintenance strategies developed in other DT domains. For example, DT frameworks trained on telemetry or usage data have been used to detect anomalies, such as signal drift or timing inconsistencies in industrial systems [78], while hospital-wide DT architectures have been proposed to model equipment performance and recommend maintenance interventions based on simulated aging profiles [79]. These cross-domain developments highlight the relevance of data-driven maintenance concepts, even though the referenced works do not pertain directly to PET or SPECT scanners. Despite this promise, clinical translation is limited by restricted access to vendor telemetry and a lack of long-term empirical datasets for medical-grade component aging. Addressing these gaps will require collaboration between industry, clinics, and regulators to define interoperable iDT standards and sharing of anonymized operational datasets.

#### Quantitative data correction and protocol optimization

AI-based reconstruction and correction methods are increasingly integrated into simulation-driven imaging environments and may function as computational modules within emerging iDT frameworks, even when they do not independently constitute fully realized DT systems. Quantitative accuracy in PET and SPECT imaging relies heavily on robust data correction methods that address multiple physical and system-dependent effects [80]. iDTs can model and optimize a wide range of quantitative corrections, including normalization, attenuation, scatter, motion correction, dead time, and partial volume correction [81–88]. By simulating scanner-specific acquisition chains, iDTs enable the evaluation and fine-tuning of these corrections under controlled conditions. For example, motion correction remains one of the most extensively explored areas of DT-enabled analysis. DT environments incorporating anthropomorphic computational phantoms (e.g., XCAT) facilitate the simulation of respiratory and cardiac motion within scanner geometries, revealing how detector misalignment and timing offsets propagate into image artifacts [89]. As highlighted by Kyme and Fulton [90] and Mohammadi et al. [91], motion artifacts remain a critical source of diagnostic uncertainty, particularly in nuclear cardiology. Differences between rotating and stationary systems further complicate correction strategies, as shown by Nichols and Van Tosh [92].

Several representative studies have explored iDT-enabled motion correction across cardiac, brain, and total-body PET and SPECT systems, highlighting improvements in quantitative accuracy and consistency across modalities [93–98]. These works encompass both acquisition-level and reconstruction-level motion compensation strategies. Notably, Blume et al. [99,100] demonstrated joint image-motion reconstruction using a B-spline motion model, implemented through a DT of the Siemens Biograph 16 PET/CT scanner, an approach that integrates the motion model directly into the reconstruction cost function and requires scanner-level DT modeling. AI-based approaches have further extended these methods toward adaptive, data-driven motion compensation frameworks within iDT environments. Similarly, iDT-based MC simulations have been applied to evaluate attenuation and scatter correction schemes [101–103], as well as partial-volume and resolution recovery methods, where iDT-based MC simulations were used to assess recovery coefficients and evaluate quantification bias across lesion sizes [104–106].

#### Dosimetry, dose optimization, and image reconstruction benchmarking

Many reconstruction and dosimetry frameworks discussed in this section rely on physics-informed simulation environments that can serve as computational components of iDT ecosystems, supporting quantitative evaluation and protocol optimization. Physics-based iDT platforms (e.g., GATE and reDoseMC) replicate the physics of detectors, collimators, and radiopharmaceutical transport, including simplified models of timing and energy response. These platforms provide a unified environment for quantitative dosimetry evaluation, dose optimization, and reconstruction benchmarking [107,108]. By explicitly modeling scatter and attenuation within realistic object and system geometries, and spatial resolution as a function of detector configuration, iDTs enable ground-truth comparisons for radionuclide therapy planning and quantitative imaging [109,110].

Complementing these simulations, reconstruction algorithms, such as xSPECT, have also been systematically evaluated for their ability to improve quantitative accuracy. Armstrong and Hoffmann [111] demonstrated that the conjugate gradient-based xSPECT reconstruction algorithm enables more reliable activity concentration quantification, while Miyaji et al. [112] validated xSPECT through both phantom and clinical evaluation in bone SPECT/CT. Similarly, Dickson et al. [113] highlighted how reconstruction methods and scanner characterization directly influence the diagnostic capability of normal databases in FP-CIT SPECT.

MC-based DTs play a central role in image reconstruction by providing physics-accurate models for forward and backward projection operators. They also generate MC-derived system matrices, sensitivity images, and collimator–detector response functions that may be employed to improve iterative statistical reconstruction in both PET and SPECT. This approach was demonstrated in foundational work by Rafecas et al. [114], who introduced a MC-based probability matrix for 3D reconstruction in the MADPET-II scanner, enabling high-fidelity modeling of detector response. Similarly, Lazaro et al. [115] implemented fully 3D MC reconstruction for SPECT, showing the feasibility of MC-based system modeling in clinical geometries. More recently, Nguyen et al. [116] developed efficient MC system modeling for preclinical pinhole SPECT, illustrating how modern computational methods, including GPU acceleration, enable practical MC-informed reconstruction pipelines.

In parallel, Sinsoilliez et al. proposed a modular DT pipeline for molecular radiotherapy dosimetry, integrating anatomical phantoms, physiologically based pharmacokinetic models, and GATE-based MC simulations to optimize absorbed dose estimation in peptide receptor radionuclide therapy. Their stepwise architecture demonstrated the feasibility of patient-specific DTs for protocol harmonization, setting the stage for multicenter validation and standardization in personalized radionuclide therapy planning [117].

Building upon these modular dosimetry frameworks, iDTs can also be extended to optimize administered activity and acquisition duration through quantitative modeling of image noise and count statistics [118–120]. In PET, MC-derived frameworks have been used to analyze the relationship between activity distribution, attenuation, and noise-equivalent count rate (NECR), enabling virtual optimization of injected dose to balance image quality, patient exposure, and scan time [121,122]. Similar principles apply to SPECT, where iDTs can simulate count-based noise propagation under different collimator–detector configurations or acquisition protocols to evaluate trade-offs between administered activity and reconstructed image quality and quantitative accuracy [123]. Such DT-assisted analyses can also support adaptive, AI-driven imaging protocols that predict the optimal combination of dose and acquisition parameters for specific tracers or patient populations [124]. Early implementations by Karakatsanis et al. [33] demonstrated the feasibility of this approach in PET, underscoring its potential for personalized and resource-efficient imaging workflows.

Collectively, these studies affirm that iDT-informed MC environments, together with advanced reconstruction algorithms and modular dosimetry pipelines, provide harmonized, reproducible, and extensible frameworks for benchmarking dosimetry and image quantification across centers, while facilitating AI-driven optimization of imaging protocols.

#### Synthetic data generation and AI training

AI-driven synthetic data generation pipelines are increasingly integrated into simulation-based imaging environments and may function as data-generation modules within broader iDT frameworks. DTs of PET and SPECT scanners are increasingly leveraged to produce synthetic datasets that reflect hardware-specific conditions, such as low-dose counts, detector misalignments, or collimator designs. These datasets provide ground truth for training AI models in tasks like denoising, attenuation correction, segmentation, and lesion detection. Unlike purely image-based synthesis, iDT-generated data retain the statistical and physical properties of detector-level acquisition, enabling more robust AI validation and potentially supporting regulatory approval pathways.

For instance, Wang et al. [125] generated synthetic PET images of synaptic density and amyloid from fluorodeoxyglucose (FDG)-PET, while Fard et al. [126] synthesized interictal SPECT from MRI and PET inputs, reducing radiation exposure in epilepsy workflows. Cross-domain translation has also been demonstrated, with Lopes et al. [127] adapting dopaminergic PET to SPECT using CycleGAN, and Watanabe et al. [128] applying generative transformers to FP-CIT SPECT images.

Synthetic datasets are also critical for benchmarking AI performance. de Bourbon et al. [129] embedded synthetic lesions into PET scans to quantify detection accuracy. Moreover, attenuation and scatter correction pipelines have been enhanced by iDT-generated datasets, with applications in total-body PET [130], ovarian cancer PET/CT [131], and myocardial perfusion SPECT [132,133]. Beyond corrections, structural MRI or CT can be translated into PET domains, as demonstrated by Rajagopal et al. [134] for whole-body FDG-PET and Salehjahromi et al. [135] in lung cancer prognosis. In hardware-limited designs, Farsani et al. [136] showed that synthetic data can enhance PET signal-to-noise ratio in short-FOV systems, offering image quality comparable to total-body scanners.

Collectively, these studies demonstrate that iDT frameworks not only expand the scope of synthetic dataset generation but also ensure that datasets remain grounded in the physical properties of scanner hardware, an essential step toward regulatory-grade AI training and validation.

#### Scanner design optimization

The use of iDTs has emerged as a powerful approach for optimizing and developing innovative PET and SPECT scanner geometries [137]. In many cases, these studies rely on advanced MC-based simulation environments that function as foundational components of iDTs, enabling virtual prototyping and system-level performance analysis even when a fully data-synchronized DT framework is not yet implemented. MC-based DT environments have long been employed to systematically explore how detector configurations, collimator designs, crystal materials, and AFOV lengths influence imaging performance metrics, such as sensitivity, spatial resolution, energy resolution, and cost efficiency [138]. Through virtual prototyping and simulation-based evaluation, iDTs enable comprehensive assessment of design trade-offs across modalities, balancing system complexity, performance, and manufacturability before physical implementation [139].

Recent iDT-based frameworks have been applied to assess the feasibility of cost-effective total-body PET systems and limited AFOV scanners optimized for low-income countries [140–142]. Simulation studies of medium-AFOV and sparse PET configurations based on flat monolithic panels have shown promising trade-offs between resolution, sensitivity, and system cost [143–146]. Comparative evaluations of sparse versus full detector geometries have demonstrated that optimized sparse configurations can retain uniform sensitivity while substantially reducing detector material and manufacturing cost [147]. In this context, flexible time-of-flight-DOI panel detectors and plastic scintillator-based J-PET designs further support the development of modular, mobile, and cost-efficient imaging systems capable of maintaining clinical-grade image quality [148,149].

Parallel advances in detector materials and timing performance have also accelerated innovation in PET instrumentation. Simulation-based investigations have explored improved Cherenkov-based time estimation and surface effects in monolithic detectors [150,151], while recent studies of high-Z Cherenkov radiators, such as TlCl:Be,I [152] and UV-enhanced SiPM readouts for BGO scintillators [153], have achieved coincidence timing resolutions below 300 ps, highlighting how DT-assisted modeling can guide material selection and timing optimization. Beyond clinical scanners, MC-based DTs have also been applied to specialized applications, such as plant imaging, where geometry optimization and positron-range correction substantially enhance quantitative accuracy [154]. Finally, recent comprehensive reviews have emphasized the expanding role of DTs in guiding the design of multifunctional and dedicated PET systems with irregular geometries for organ-specific and cost-efficient imaging [155].

**Fig. 3.**
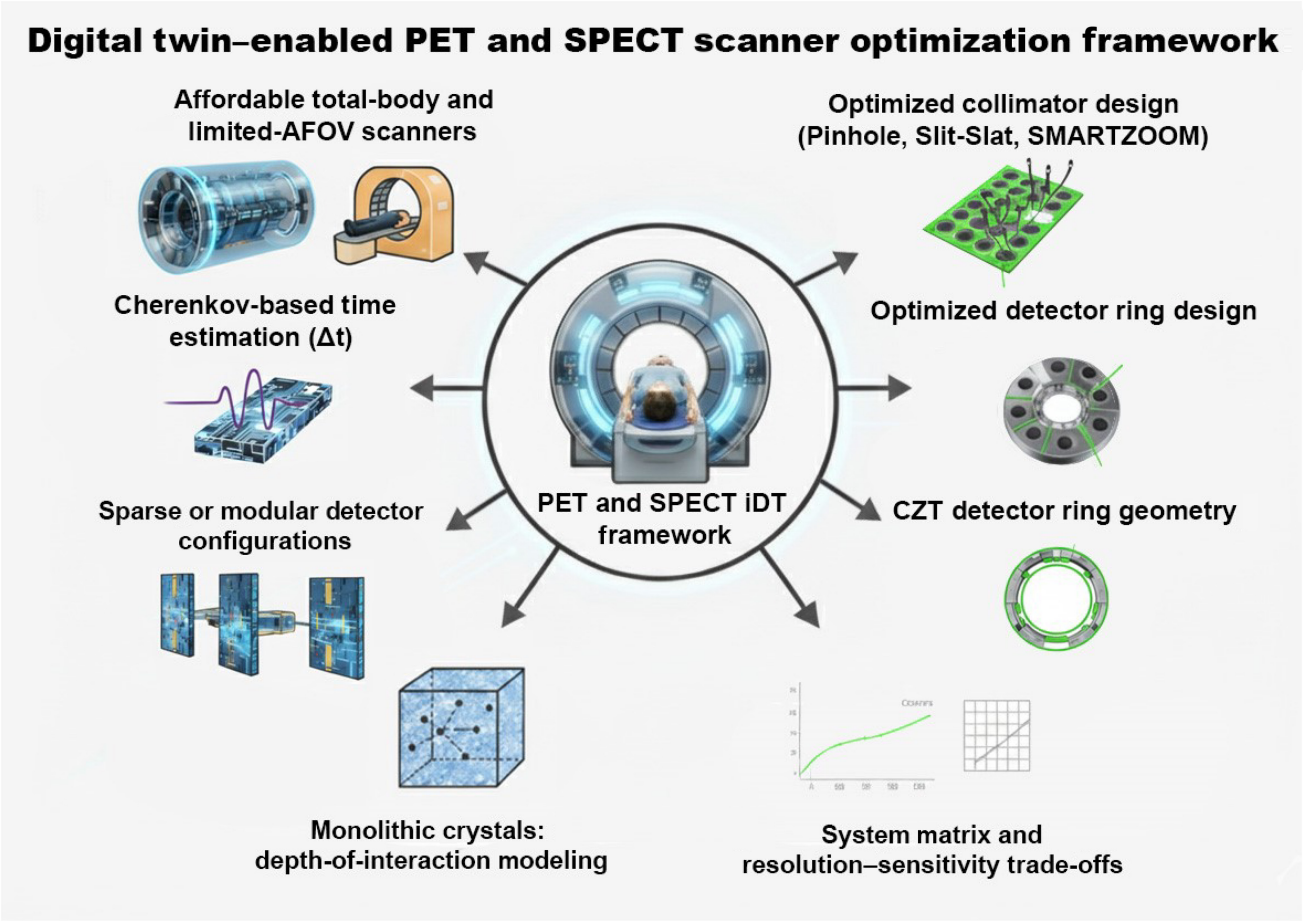
Schematic representation of a digital twin-enabled PET and SPECT scanner optimization framework, illustrating how iDTs can support design and performance optimization across components, such as collimators, detector rings, and system matrices.

In SPECT, MC-based iDT frameworks have been widely used to optimize collimator–detector geometry and novel system designs. Multi-pinhole brain and small-animal SPECT systems have demonstrated substantial sensitivity and resolution improvements through optimization of pinhole geometry, including number, diameter, and acceptance angles, relative to LEHR or fan-beam collimation [156–161]. These simulation-based frameworks have also been extended to specialized configurations, such as breast-dedicated and synthetic compound-eye designs, highlighting the flexibility of iDT approaches in developing application-specific geometries [162,163]. Other design-oriented studies have evaluated the performance of alternative collimator types and detector materials to further enhance image quality and system sensitivity [164,165]. IQ-SPECT (cardiac) leverages SMARTZOOM converging collimators and cardio-centric orbits to achieve markedly faster acquisitions; MC modeling of its multifocal geometry has been developed to evaluate performance and protocol trade-offs [166,167]. Emerging 360° CZT ring SPECT/CT systems (e.g., VERITON/StarGuide) illustrate how digital prototypes guide ring geometry and energy-window choices for isotopes, such as ^99m^Tc and ^177^Lu, balancing sensitivity with scatter/penetration constraints [168–170]. Beyond pinhole and converging designs, slit-slat configurations have been optimized (analytically and via MC) for resolution–sensitivity trade-offs and calibrated system matrices for reconstruction, underscoring SPECT-specific design opportunities [71,171,172]. Taken together, these studies demonstrate the versatility of iDTs as a unifying framework for hardware innovation in both PET and SPECT. By serving as a digital testbed that bridges simulation and physical realization, iDTs accelerate scanner development, guide geometry selection, and minimize reliance on costly iterative prototyping. As such, scanner design optimization through iDTs represents a cornerstone in advancing the next generation of cost-effective and high-performance nuclear imaging instrumentation. A schematic representation of PET and SPECT iDT optimization framework is presented in Fig. 3, illustrating how iDTs support scanner design and performance optimization across components, such as collimators, detector rings, detector materials, and system matrices.

Table 1 provides an integrated comparative overview of representative studies across all 6 iDT application domains discussed in the “Nuclear medicine: PET and SPECT imaging” section, including (a) scanner calibration and performance optimization; (b) predictive maintenance and fault detection; (c) quantitative data correction and protocol optimization; (d) dosimetry, injected dose optimization, and reconstruction benchmarking; (e) synthetic data generation and AI training; and (f) scanner design optimization. Each study summarizes the system scope, modeling tools, hardware focus, key limitations, analytical contribution, and the DT maturity/synchronization level, illustrating how different levels of DT development—from simulation-based frameworks to validated digital replicas and hybrid AI–physics prototypes—have been leveraged to address instrumentation-level challenges across nuclear imaging. This classification also highlights that many existing implementations remain at the level of simulation-based digital replicas or hybrid computational frameworks, while fully synchronized iDTs remain an emerging direction. While several implementations remain primarily simulation-based or vendor-specific prototypes rather than fully realized DTs, these studies provide essential building blocks for the development of future iDT ecosystems by enabling physics-informed design exploration and system-level optimization.

**Table 1.** Comparative summary of representative studies implementing instrumentation digital twin (iDT)-related frameworks for positron emission tomography (PET) and single-photon emission computed tomography (SPECT) imaging systems, organized by key application categories. The table highlights application domain, modeling tools, hardware focus, key limitations, and the digital twin maturity or synchronization level of each study.

Study	Modality	Category	DT scope/Purpose	Digital twin maturity/Synchronization level	Tools used	Hardware focus	Key limitations	Analytical contribution
Pommranz et al. [8]	PET/CT (LAFOV Biograph Vision Quadra)	Calibration and performance	System-level DT for simulation, motion modeling, dosimetry, AI training	Validated digital replica	GATE, Siemens e7 tools, root2lm	Extended axial FOV, detector normalization	Vendor-specific, limited reproducibility	Experimentally validated full-system PET DT
Peña-Acosta et al. [27]	PET/CT (Vision and Quadra)	Calibration and performance	Verification of GATE models via NEMA NU2–2018 protocols	Validated digital replica	GATE, CASToR, STIR	Calibration fidelity, axial gaps	No patient modeling, limited to NEMA	Open-source validation of PET DT models
Lorduy-Alós et al. [107]	PET (Biograph Vision)	Dosimetry and reconstruction	Hardware-informed voxel-level dosimetry and image reconstruction	Simulation-based framework	GATE, CASToR, 3DSlicer	Phantom-based dose estimation, detector physics	Focused on prostate, no dynamic modeling	Voxel-level personalized dosimetry framework
Laurent et al. [230]	PET (Vision Quadra)	Calibration and reconstruction	DL-based scatter estimation for LAFOV	Hybrid AI–physics prototype	GATE, CNN (U-Net)	Scatter in extended FOV	Limited to scatter sinogram, no full DT	DL-based scatter correction in PET simulation framework
Sanz-Sanchez et al. [231]	PET	Synthetic data and AI integration	Synthetic PET datasets for CNN training	Simulation-based framework	GATE, CASToR, Python	Low-dose, artifact injection	No real PET validation	Synthetic PET dataset generation pipeline
Li et al. [222]	177Lu SPECT	Dosimetry and reconstruction	CNN-based deblurring for dose map	Hybrid AI–physics prototype	DVK, DPM MC, U-Net	Low-resolution SPECT hardware	Phantom validation only	AI-assisted voxel dosimetry improvement
Wikberg et al. [223]	177Lu SPECT	Synthetic data/Dosimetry	Sparse SPECT → synthetic projections	Hybrid AI–physics prototype	MC-OSEM, SARec, DL U-Net	Projection reduction	Slight marrow dose degradation	Sparse-acquisition SPECT reconstruction framework
Bui et al. [232]	SPECT	Quantitative data correction and protocol optimization	Translational motion correction (eDCC)	Simulation-based framework	GATE, Symbia SPECT/CT	Rigid-only motion	Simplified assumptions	Data-driven motion correction method
Karakatsanis et al. [233]	PET	PET scan protocol optimization	WB dynamic PET and parametric imaging protocols optimization	Simulation-based framework	GATE, STIR	Effect on data SNR, and parametric image quality of scan protocol and type of parameter estimation method	Limited to a specific human torso phantom, scanner model and tracer-organ kinetic properties	Determination of optimal number of WB passes and bed frame duration for dynamic WB scan protocols of limited axial FOV
Zhang et al. [234]	SPECT-MPI	Quantitative data correction and protocol optimization	DL correction for perfusion defect detection	Hybrid AI–physics prototype	VoxelMorph, QPS, QGS	Cardiac motion	No angiography validation	DL-based cardiac motion correction
Auer et al. [235]	SPECT (brain)	Calibration and design	STL-based modeling of AdaptiSPECT-C	Simulation-based framework	GATE, SolidWorks STL, XCAT	Detector/collimator design	Complex mesh modeling, no patient data	Full-system STL-based SPECT simulation model
Jia et al. [236]	90Y SPECT	Dosimetry and reconstruction	End-to-end DL scatter correction and voxel dosimetry	Hybrid AI–physics prototype	CNN, DblurDoseNet, MC (SIMIND)	Scatter/attenuation hardware	Limited to liver RE	DL-based scatter correction for voxel dosimetry
Vandenberghe et al. [141]	PET	Scanner design	Simulation of medium-cost long AFOV PET with modular detectors	Simulation-based framework	GATE, CASToR	Sparse ring LAFOV geometry	Cost-focused, not validated clinically	Feasibility study of cost-efficient LAFOV PET
Chen et al. [156]	SPECT (brain)	Scanner design	Optimized collimator geometry for high-sensitivity brain SPECT	Simulation-based framework	GATE, MATLAB	Multi-pinhole SPECT	Phantom-based	Collimator design optimization via simulation

LAFOV, long axial field of view; DT, digital twin; AI, artificial intelligence; FOV, field of view; CNN, convolutional neural network; MC, Monte Carlo; OSEM, ordered subset expectation maximization; AFOV, axial field of view; GATE, Geant4 Application for Tomographic Emission

## Challenges in iDTs for PET and SPECT Imaging

**Fig. 4.**
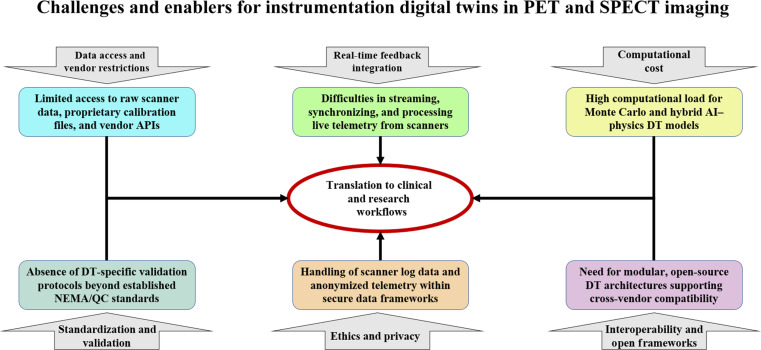
Key challenges limiting the implementation and clinical translation of iDTs in PET and SPECT imaging systems. The figure illustrates major barriers limiting the clinical adoption of iDTs, including restricted access to vendor-specific scanner data, high computational cost of physics-based models, lack of standardized validation frameworks, ethical/privacy concerns, and the absence of real-time feedback integration. These limitations collectively hinder the translation of iDTs from research prototypes to clinically actionable tools.

Despite recent advances, current iDT implementations in PET and SPECT remain fragmented and task-specific. Many approaches focus on motion correction, dosimetry, or synthetic data generation in isolation, but fail to capture the full imaging chain from detector physics to reconstruction. Similarly, the absence of real-time feedback and vendor-neutral interoperability limits clinical translation. While Fig. 4 outlines the 6 key challenges that currently limit the implementation and clinical adoption of iDTs, these challenges naturally translate into a set of technical gaps and development opportunities that must be addressed to advance the field. To contextualize these limitations within a forward-looking framework, a summary table in the “Roadmap and Future Directions” section maps 9 major gaps in each challenge to specific research and infrastructure needs, highlighting where progress in hardware modeling, data access, interoperability, and real-time integration can enable more mature and clinically deployable iDT ecosystems.

### Data access and hardware fidelity

Vendor restrictions continue to limit access to raw detector, collimator, and electronics data, creating substantial barriers to developing high-fidelity iDT models. In many cases, the lack of transparency forces researchers to rely on reverse-engineering approaches to infer detector efficiency variations, normalization factors, and random estimates, an issue documented, for example, in studies attempting to reconstruct proprietary PET system characteristics [173]. Likewise, the absence of detailed descriptions of detector misalignment, gain drift, and component aging undermines the realism of calibration and long-term performance simulations. These constraints have been highlighted in multicenter LAFOV PET evaluations, where vendor-specific tools have resulted in limited reproducibility across sites [174,175]. Furthermore, the integration of dose-optimization frameworks into iDT architectures remains hindered by the lack of standardized reference protocols and cross-vendor calibration datasets. Addressing these limitations will require broader access to hardware specifications, the establishment of computer-aided design (CAD)-to-simulation pipelines, and the development of probabilistic reliability models, such as fault-tree and event-tree analysis, to realistically capture detector behavior throughout the scanner life cycle.

### Real-time feedback and system integration

Most current iDT implementations operate in an offline and static manner, limiting their ability to adapt to changing scanner conditions or patient-specific variations. The absence of closed-loop architectures, where live scanner telemetry continuously updates the DT, prevents the development of adaptive calibration strategies and online correction workflows. Although the integration of real-time detector signals, environmental sensors, and edge-AI processing holds substantial promise for enabling predictive maintenance and continuous quality assurance, restricted access to telemetry data remains a critical barrier. As DTs advance toward tighter coupling with physical imaging systems, real-time feedback mechanisms could support adaptive acquisition control, dynamic protocol optimization, online QA, and early hardware fault detection. These capabilities underscore the opportunity for fully closed-loop DT frameworks that synchronize with live data streams to enable intelligent, self-correcting imaging workflows.

### Physics-based modeling vs. computational cost

Physics-based MC simulations, such as those performed using GATE, provide high-fidelity modeling of detector physics, scatter, attenuation, and system response. However, their substantial computational cost limits their practicality for real-time or adaptive applications, including online calibration and motion correction. Recent advances in GPU-based MC platforms and high-throughput central processing unit (CPU) implementations have begun to mitigate these limitations, with GPU-accelerated MC scatter-correction methods for PET/MRI [176] and LAFOV PET [177], together with broader reviews of GPU MC engines [53], fast CPU-based PET simulation [178], and emerging frameworks, such as ultrafast Monte Carlo positron emission tomography simulator [54], demonstrating that modern computational strategies can substantially reduce simulation times and bring MC-driven iDT applications closer to real-time feasibility. In practical implementations, CPU-based MC simulations of PET and SPECT acquisition chains may require several hours to days depending on scanner geometry, number of simulated events, and reconstruction complexity, as reported in multiple PET or SPECT simulation studies [56,179,180]. Recent GPU-accelerated MC frameworks have demonstrated order-of-magnitude speed improvements, often achieving approximately 10× to 100× reductions in simulation time [181,182]. However, MC-derived system matrices and detector response models can impose substantial memory requirements, as high-resolution PET system matrices may reach effective sizes on the order of 10^12^ elements, creating significant storage and scalability challenges for large-scale or LAFOV systems [183]. Representative computational strategies and their reported performance characteristics are summarized in Table 2, based on acceleration ranges commonly reported in PET and SPECT MC simulation and GPU-accelerated imaging studies. Physics-based MC frameworks remain the most accurate approach for modeling detector physics, photon transport, and system geometry in instrumentation digital replicas of PET and SPECT systems, offering high physical fidelity and well-established validation methodologies [184–186]. However, these models are computationally intensive and often require substantial processing time, particularly for large-scale or patient-specific simulations and LAFOV systems [8]. In contrast, hybrid AI–MC pipelines increasingly combine physics-informed simulation with data-driven models to accelerate reconstruction, correction, and protocol optimization tasks [187,188]. While such approaches significantly improve computational scalability and enable near-real-time inference, they may introduce limitations in physical interpretability and require extensive training datasets and validation. Consequently, emerging iDT ecosystems increasingly adopt hybrid architectures that leverage the complementary strengths of physics-based modeling for accuracy and AI-driven methods for computational efficiency and scalability. These constraints have motivated the development of hybrid frameworks that couple physics-driven simulations with AI-based surrogate models to accelerate inference without sacrificing accuracy. Recent examples include efficient collimator–detector modeling pipelines [189] and generative adversarial network-based fast dosimetry frameworks [190], both demonstrating how learned surrogates can approximate computationally intensive components of MC workflows. Such hybrid physics–AI approaches represent an important opportunity for enabling near-real-time optimization, reducing simulation overhead, and supporting the deployment of adaptive iDT architectures.

**Table 2.** Overview of representative computational strategies used in instrumentation digital twin (iDT) frameworks for positron emission tomography (PET) and single-photon emission computed tomography (SPECT) imaging. The table summarizes modeling approaches, typical computational characteristics, and approximate acceleration factors reported in the literature for Monte Carlo-based and artificial intelligence (AI)-assisted simulation frameworks.

Computational approach	Modeling strategy	Typical acceleration	Advantages	Limitations
CPU-based Monte Carlo	Full physics-based photon transport simulation	Baseline (hours–days runtime depending on system complexity)	High physical fidelity and accurate detector modeling	Computationally expensive
GPU-accelerated Monte Carlo	Parallelized photon transport on GPUs	~10×−100× speedup	Significant runtime reduction and improved scalability	Hardware dependence
Hybrid AI–Monte Carlo	Physics simulation combined with learned surrogate models	Potential near real-time inference in specific tasks	Balance between accuracy and computational efficiency	Requires training datasets and validation
AI surrogate models	Data-driven approximations of physics components	Orders-of-magnitude acceleration	Fast inference and scalable deployment	Reduced physical interpretability

CPU, central processing unit; AI, artificial intelligence

### Validation and standardization

A major barrier to the broader adoption of iDTs is the absence of consensus standards for validating iDT fidelity across scanners, vendors, and clinical institutions. Regulatory agencies, such as the Food and Drug Administration and European Medicines Agency emphasize reproducibility and cross-site harmonization, meaning that iDT-based models must demonstrate consistent performance under diverse acquisition conditions before gaining clinical acceptance. Findings from multicenter quantitative SPECT initiatives, including MRTDosimetry, have already underscored the value of standardized calibration protocols and shared reference datasets to ensure reliability across sites [109]. These observations highlight the need for coordinated multicenter efforts, open-source validation frameworks, and harmonized performance metrics to establish robust benchmarks for iDT accuracy and generalizability.

In practice, the validation of iDTs will likely follow a multistage hierarchy [191]. Early-stage research frameworks typically rely on simulation validation and phantom experiments to verify physical modeling accuracy and reconstruction performance [192]. For clinical deployment, regulatory-grade iDT systems would likely require prospective validation in real-world clinical workflows, demonstrating consistent performance across scanners, patient populations, and acquisition conditions, a requirement for achieving regulatory approval and enabling the safe integration of iDT technologies into clinical imaging practice [193–195]. In this context, it is important to distinguish between research-oriented simulation environments used for methodological development and regulatory-grade decision-support systems that may influence clinical decision-making. A practical validation pathway for iDTs may therefore follow a staged cascade [196,197]. Initial development stages typically involve physics verification using digital phantoms and controlled simulation experiments to ensure accurate modeling of detector physics and acquisition chains. This is followed by phantom-based benchmarking and cross-center reproducibility studies using standardized acquisition protocols and shared reference datasets adopting standardized open and vendor-agnostic data formats even at the raw data (list-mode and sinograms) level [198]. Subsequent stages may include retrospective validation on multi-institutional multimodality clinical datasets to evaluate robustness across patient populations and scanner configurations. Ultimately, prospective validation within real clinical workflows would be required before iDT systems could support clinical decision-making [199]. In this context, emerging regulatory guidance frameworks, such as good machine learning practice and model-informed device development, provide useful conceptual foundations for integrating data-driven models and simulation-based systems into regulated medical device development pathways [200–203].

### Ethical, privacy, and cybersecurity concerns

iDTs inevitably handle sensitive patient information, device telemetry, and system-level operational data, raising important concerns related to privacy, data governance, and cybersecurity. Ensuring compliance with regulatory frameworks, such as the Health Insurance Portability and Accountability Act and the General Data Protection Regulation, remains essential, yet this area is still insufficiently explored within current iDT implementations. As DTs become increasingly integrated with clinical workflows, secure mechanisms for data transfer, anonymization, and access control will be required to protect both patient identity and proprietary scanner information. As iDTs become increasingly integrated with physical imaging systems, cybersecurity risks associated with cyber-physical infrastructures must also be considered [204,205]. Potential vulnerabilities include unauthorized access to scanner telemetry streams, interception of system-level operational data, model tampering, and adversarial attacks targeting AI components embedded within hybrid DT architectures [206–208]. Such risks could potentially affect calibration workflows, automated correction pipelines, or predictive maintenance models if adequate security mechanisms are not implemented [209]. In multicenter DT development, privacy-preserving collaborative strategies, such as federated learning, may play an important role by enabling distributed institutions to jointly train AI models without directly sharing sensitive imaging data or proprietary scanner information [210]. Federated learning frameworks therefore support decentralized model development while preserving data locality, facilitating the relatively more secure and scalable development of iDT ecosystems across institutions [211]. However, federated learning alone may not fully prevent information leakage from shared updates unless combined with controls such as secure aggregation and other privacy-enhancing techniques [212]. At the same time, the use of privacy-preserving synthetic datasets generated within iDT environments offers a promising pathway for enabling AI development and algorithm benchmarking without exposing protected health information [129]. These considerations collectively highlight the need for robust security architectures, standardized data-sharing protocols, and privacy-preserving approaches that ensure trust and regulatory readiness in future iDT ecosystems.

While technical security mechanisms can mitigate specific vulnerabilities, the safe deployment of iDTs ultimately requires well-defined cybersecurity architectures and governance frameworks [213–215]. These should include secure telemetry pipelines, robust authentication and access control mechanisms, model integrity verification, and institutional policies governing data access, model updates, and system monitoring [216–219]. Establishing such governance structures will be essential to ensure safe deployment of DT technologies within clinical imaging infrastructures while maintaining trust, transparency, and regulatory compliance [220,221].

### AI dataset generation and rare scenarios

Despite their growing use, many existing synthetic data pipelines fail to capture the full diversity of clinical imaging scenarios, particularly rare pathologies, subtle anatomical variations, realistic artifacts, and variations in acquisition conditions. These limitations can lead to AI models that generalize poorly outside training distributions. Recent studies embedding synthetic lesions into PET datasets [129] or performing cross-modality PET-to-SPECT translations [125] demonstrate how iDT-generated datasets can enhance AI robustness by incorporating hardware-faithful signal characteristics and controlled variations in imaging conditions. Such approaches also provide structured pathways for regulatory validation by enabling reproducible testing across diverse scenarios. Moving forward, leveraging iDT frameworks to generate physically grounded, diverse, and clinically representative datasets offers a critical opportunity to support reliable AI development and stress-test model performance under rare or adverse conditions, and promote the creation of regulatory-grade validation resources.

**Fig. 5.**
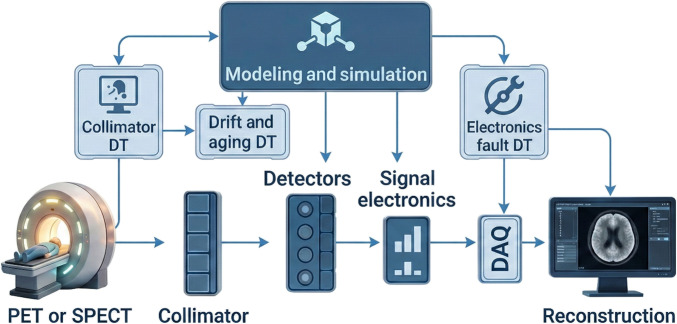
Schematic representation of iDT integration in PET and SPECT imaging systems. The diagram illustrates how DTs can model key hardware components, including collimators, detectors, and signal electronics, to capture effects, such as detector gain drift, alignment errors, collimator-geometry variations, and electronic faults. Note that the physical collimator element corresponds specifically to SPECT, whereas PET relies on electronic collimation through coincidence detection; the figure is intended as a unified conceptual overview. These models feed into simulation and reconstruction pipelines, enabling improved calibration, predictive maintenance, and artifact correction. By embedding DTs across the acquisition chain, hardware fidelity and clinical reliability are enhanced.

Collectively, these challenges underscore that the future of iDTs in nuclear medicine lies in hybrid, modular, and validated frameworks. Hardware realism (detectors, collimators, and electronics), dynamic synchronization with scanner telemetry, and cross-center validation are not solely optional add-ons but may become prerequisites for clinical acceptance. Addressing these areas will require interdisciplinary collaboration, regulatory alignment, and open-source infrastructures, bridging the gap between current simulation prototypes and clinically actionable iDT systems. To visualize these hardware-focused gaps, Fig. 5 schematically illustrates how DTs can be embedded into the instrumentation chain, linking detectors, collimators, and electronics with their digital counterparts.

## Roadmap and Future Directions

**Fig. 6.**
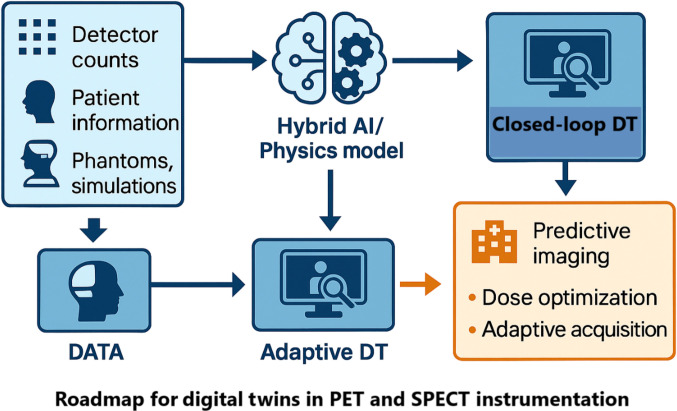
Development roadmap illustrating the evolution of iDTs from data-driven models to adaptive and closed-loop DT systems in PET and SPECT imaging. The framework integrates input data—including detector counts, patient information, digital phantoms, and Monte Carlo simulations—into hybrid AI–physics models. These models enable adaptive digital twins that update their parameters using incoming data to support predictive imaging, dose optimization, and protocol adaptation. In more advanced implementations, closed-loop digital twins operate in continuous synchronization with the physical scanner, enabling online calibration, adaptive acquisition control, and intelligent feedback during clinical imaging. The roadmap highlights the transition from static or hybrid models toward fully intelligent, closed-loop digital twin ecosystems in nuclear medicine.

Building upon the challenges identified in Fig. 4 and gaps outlined in Table 3, the next step for iDTs in PET and SPECT is to transition from isolated prototypes toward robust, hardware-level frameworks that integrate seamlessly into clinical imaging pipelines. As iDTs evolve toward clinically deployable systems, accelerating the underlying computational models becomes essential. Recent advances in GPU-based MC engines and AI-accelerated surrogate MC models have demonstrated substantial reductions in simulation time, thereby enabling near real-time modeling of scatter, attenuation, and detector response. In particular, hybrid AI–MC approaches, such as those reviewed by Sarrut et al. [188], illustrate how learned models can reproduce MC accuracy at a fraction of the computational cost, thus forming a critical technological foundation for scalable, clinically viable iDT frameworks. Figure 6 illustrates a conceptual roadmap where hardware-informed inputs are processed through hybrid iDT models, yielding outputs that directly support scanner calibration, adaptive imaging, and clinical decision-making.

**Table 3.** Highlights for 9 major gaps in current instrumentation digital twin (iDT) implementations together with corresponding opportunities for development, ranging from hardware-level modeling to real-time feedback integration

Gap area	Current limitation	Technical requirements	Opportunity for development	Expected benefits	Priority level	Timeline feasibility
Scanner hardware modeling	Lack of detailed models for detectors, collimators, and electronics; detector misalignment and coordinate inaccuracies often overlooked, leading to discrepancies between simulated and real system performance	High-fidelity component-level simulation, CAD-GDML integration, probabilistic reliability models (fault-tree, event-tree), and explicit modeling of detector coordinate and efficiency variations	Build full-system DTs that not only model detector gain drift and component aging but also identify and correct detector misalignments—critical for small gantries such as small-animal PET	Enables virtual calibration, predictive maintenance, and detection of subtle misalignments that degrade resolution; improves fidelity between simulated and real performance	High	Mid-term
Real-time feedback loop	Limited dynamic update of DTs with live scanner or AI-inferred feedback data	Real-time telemetry access; edge-AI for fast processing	Closed-loop iDTs with adaptive calibration, online QA, and AI-assisted fault prediction	Supports real-time fault detection, protocol adaptation, improved system stability	High	Short-term
Workflow integration	DTs often limited to isolated tasks (motion, dosimetry)	Modular and interoperable DT architectures	Develop scalable ecosystems linking simulation, reconstruction, and decision support, including dose management and adaptive acquisition planning	Streamlined clinical integration; improved reproducibility	Medium	Mid-term
Patient-specific iDT simulation	Reliance on static/generic phantoms	Patient-specific voxel phantoms; kinetic modeling	Personalized DTs incorporating anatomy, tracer kinetics, and motion	Higher diagnostic precision; improved therapy planning	High	Short-term
Dose optimization and NECR modeling	Current DT frameworks lack standardized dose modeling across tracers and modalities; limited integration with patient-specific activity modeling and clinical NECR data	Coupled iDT-dosimetry architectures, NECR-based dose-time simulations, cross-modality dose harmonization	Develop adaptive, AI-assisted dose optimization modules within iDTs for PET and SPECT	Enables personalized activity planning, reduced patient exposure, and harmonized multi-center imaging protocols	High	Mid-term
Scanner design optimization	Current DT implementations focus primarily on existing system replication rather than novel geometry design or component co-optimization; limited integration with manufacturing constraints and cost models	Coupled CAD-MC frameworks, AI-driven geometry exploration, and performance-cost multiobjective optimization	Develop iDT-guided scanner design ecosystems linking simulation, mechanical design, and AI-assisted optimization for PET and SPECT	Accelerates innovation, reduces prototyping costs, and enables rapid evaluation of novel geometries (e.g., sparse, modular, or 360° ring designs)	High	Mid-term
Multimodal imaging	Limited DT frameworks for PET/MR or SPECT/CT	Cross-modality registration; shared coordinates	Enable cosimulation of multimodal scans	Better synergy between modalities; unified quantitative analysis	Medium	Long-term
Synthetic data generation and AI training	Synthetic datasets often lack rare pathologies or realistic scanner-specific artifacts	Domain adaptation; GAN/Transformer-based augmentation	Use iDTs to generate diverse, hardware-faithful synthetic datasets	Enhances AI robustness and regulatory validation	High	Short-term
Standardization and sharing	No open standards for DT validation/sharing	Open-source platforms; regulatory-compliant formats	Define APIs and standard formats for multicenter DT exchange	Improves reproducibility, accelerates regulatory approval	Medium	Mid-term

GDML, Geometry Description Markup Language; DT, digital twin; PET, positron emission tomography; QA, quality assurance; NECR, noise-equivalent count rate; SPECT, single-photon emission computed tomography; CAD, computer-aided design; MC, Monte Carlo; MR, magnetic resonance; CT, computed tomography; GAN, generative adversarial network; API, application programming interface

### iDT framework proposal

A hardware-oriented iDT for nuclear medicine imaging should integrate 3 essential components: the input data streams, the core modeling engine, and the resulting predictive outputs. The inputs typically include detector signals (such as counts, timing, and energy), calibration data, anthropomorphic or digital phantoms, and simulation platforms, such as GATE. These inputs provide the physical and statistical foundation upon which the digital replica of the scanner is built.

The core iDT model typically relies on hybrid architectures that combine physics-based simulations with AI-based surrogate models. Physics-based components may include MC transport modeling and CAD-derived detector geometries. In practice, this often includes deep learning models, such as convolutional neural networks and transformer architectures. These models help improve computational efficiency while maintaining high modeling fidelity [189,190,222–226]. The outputs of such an iDT framework encompass predictive imaging performance metrics, patient-specific dose optimization, fault-detection alerts, and adaptive acquisition recommendations. These outputs enable the system to provide actionable, clinically meaningful insights. Such an iDT framework can enable closed-loop DTs in which live telemetry continuously updates the digital replica. This capability supports both research applications and real-time clinical workflows.

### Research priorities

To fully realize a robust and clinically meaningful iDT framework, several research priorities must be addressed across simulation, modeling, data acquisition, and system integration. First, enhanced simulation platforms are required to accurately capture real-world scanner behavior. In addition to improving physical fidelity, accelerating MC simulations, through GPU-based engines or AI-supported surrogate models, remains a central research priority for enabling scalable and clinically usable iDT frameworks. This includes modeling detector misalignment, gain drift, collimator imperfections, and electronic-chain variations, all of which influence quantitative fidelity. Equally important is the standardization of CAD-to-Geometry Description Markup Language (GDML) pipelines to ensure consistent and precise hardware geometry integration across simulation environments [227–229].

A second priority involves advancing AI- and physics-informed hybrid modeling. Developing surrogate models that accelerate MC-based simulations, while preserving high fidelity, remains essential for scalable iDT deployment [189,190]. In parallel, embedding uncertainty quantification into these surrogate models will be critical to ensure that iDT-generated predictions can be trusted in clinical decision-making contexts. A third priority is the establishment of multicenter datasets and validation frameworks. Shared, open-source DT infrastructures are needed to enable reproducible and transparent benchmarking across institutions [109]. Beyond this, hardware-level DT predictions must be validated against experimental measurements from scanners produced by different vendors and deployed across diverse clinical sites [174,175], ensuring generalizability and clinical robustness.

Finally, meaningful progress will require deeper integration of iDTs into clinical workflows. This includes enabling communication between iDT modules and scanner control software, via standardized application programming interfaces, as well as interoperability with PACS (Picture Archiving and Communication System), RIS (Radiology Information System), and HIS (Hospital Information System) systems. Such integration will facilitate the exploration of closed-loop applications, including real-time motion correction [95,96], adaptive tracer dosing, and online quality assurance, ultimately supporting intelligent, responsive imaging systems that evolve in tandem with patient and scanner conditions.

### Potential clinical impact

If fully realized, hardware-level iDTs have the potential to reshape multiple aspects of nuclear medicine practice. One important impact lies in the enhancement of image quality: by generating high-fidelity reconstruction elements, including system matrices, forward and backward projectors, and MC-derived scatter estimates, iDTs introduce more accurate physical modeling into the reconstruction process, leading to improved resolution, reduced artifacts, and more reliable quantitative performance. One major opportunity lies in patient-specific dose optimization, where virtual dose–response simulations could guide safer and more efficient tracer administration protocols tailored to individual physiology. In parallel, accelerated acquisition strategies informed by iDT predictions may reduce scan duration while preserving diagnostic image quality, thereby improving patient comfort and increasing scanner throughput. Hardware-centric iDTs could also drive advances in detector design and maintenance, enabling virtual prototyping of emerging technologies, such as advanced CZT detectors, multi-pinhole collimators, or self-collimating system architectures [227–229], well before physical implementation. By modeling component wear, misalignment, or degradation, these frameworks additionally support early fault detection and predictive maintenance, ultimately minimizing system downtime and enhancing operational reliability. A further transformative opportunity is the cross-modality synergy enabled by iDTs, allowing PET systems to be cosimulated with CT, MRI, or SPECT components to harmonize hybrid protocols, reduce intermodality variability, and optimize overall radiation exposure [226].

## Conclusion

iDT technology has the potential to transform PET and SPECT imaging by creating high-fidelity virtual replicas of scanners that support calibration, optimization, and innovation beyond the limits of physical hardware, as well as in advance of system construction. This review highlighted how iDTs have been applied to hardware-level tasks, such as detector calibration, quantitative correction and protocol optimization, dosimetry benchmarking, synthetic dataset generation, and scanner design optimization.

Despite these advances, current implementations remain fragmented, often limited to isolated applications without full modeling of detectors, collimators, or electronic components, and with little integration of real-time feedback or multimodal workflows. Addressing these gaps will require modular, physics-informed, and AI-enhanced iDT frameworks, validated across centers and supported by open, vendor-neutral platforms.

The path forward will also depend on interdisciplinary collaboration between physicists, engineers, computer scientists, and clinicians as well as industry, academics, and clinical practice. By combining domain expertise with shared infrastructures and efficient clinical trials, iDTs can evolve from research prototypes into reliable clinical tools, enabling smarter, adaptive, harmonized, standardized and patient-centered nuclear imaging systems.
